# No Pairwise Interactions of GmSNAP18, GmSHMT08 and AtPR1 with Suppressed *AtPR1* Expression Enhance the Susceptibility of Arabidopsis to Beet Cyst Nematode

**DOI:** 10.3390/plants12244118

**Published:** 2023-12-09

**Authors:** Liuping Zhang, Jie Zhao, Lingan Kong, Wenkun Huang, Huan Peng, Deliang Peng, Khalid Meksem, Shiming Liu

**Affiliations:** 1 State Key Laboratory for Biology of Plant Diseases and Insect Pests, Institute of Plant Protection, Chinese Academy of Agricultural Sciences, Beijing 100193, China; liupingz2013@163.com (L.Z.); zhaojie_h@126.com (J.Z.); konglingan@caas.cn (L.K.); wkhuang2002@163.com (W.H.); penghuan@caas.cn (H.P.); pengdeliang@caas.cn (D.P.); 2 Department of Plant, Soil and Agricultural Systems, Southern Illinois University, Carbondale, IL 62901, USA; meksem@siu.edu

**Keywords:** Arabidopsis, α-SNAPs, SHMTs, AtPR1, beet cyst nematode, susceptibility

## Abstract

*GmSNAP18* and *GmSHMT08* are two major genes conferring soybean cyst nematode (SCN) resistance in soybean. Overexpression of either of these two soybean genes would enhance the susceptibility of Arabidopsis to beet cyst nematode (BCN), while overexpression of either of their corresponding orthologs in Arabidopsis, *AtSNAP2* and *AtSHMT4*, would suppress it. However, the mechanism by which these two pairs of orthologous genes boost or inhibit BCN susceptibility of Arabidopsis still remains elusive. In this study, Arabidopsis with simultaneously overexpressed *GmSNAP18* and *GmSHMT0* suppressed the growth of underground as well as above-ground parts of plants. Furthermore, Arabidopsis that simultaneously overexpressed *GmSNAP18* and *GmSHMT08* substantially stimulated BCN susceptibility and remarkably suppressed expression of *AtPR1* in the salicylic acid signaling pathway. However, simultaneous overexpression of *GmSNAP18* and *GmSHMT08* did not impact the expression of *AtJAR1* and *AtHEL1* in the jasmonic acid and ethylene signaling pathways. GmSNAP18, GmSHMT08, and a pathogenesis-related (PR) protein, GmPR08-Bet VI, in soybean, and AtSNAP2, AtSHMT4, and AtPR1 in Arabidopsis could interact pair-wisely for mediating SCN and BCN resistance in soybean and Arabidopsis, respectively. Both AtSNAP2 and AtPR1 were localized on the plasma membrane, and AtSHMT4 was localized both on the plasma membrane and in the nucleus of cells. Nevertheless, after interactions, AtSNAP2 and AtPR1 could partially translocate into the cell nucleus. GmSNAP18 interacted with AtSHMT4, and GmSHMT4 interacted with AtSNAP2. However, neither GmSNAP18 nor GmSHMT08 interacted with AtPR1. Thus, no pairwise interactions among α-SNAPs, SHMTs, and AtPR1 occurred in Arabidopsis overexpressing either *GmSNAP18* or *GmSHMT08*, or both of them. Transgenic Arabidopsis overexpressing either *GmSNAP18* or *GmSHMT08* substantially suppressed *AtPR1* expression, while transgenic Arabidopsis overexpressing either *AtSNAP2* or *AtSHMT4* remarkably enhanced it. Taken together, no pairwise interactions of GmSNAP18, GmSHMT08, and AtPR1 with suppressed expression of *AtPR1* enhanced BCN susceptibility in Arabidopsis. This study may provide a clue that nematode-resistant or -susceptible functions of plant genes likely depend on both hosts and nematode species.

## 1. Introduction

Plant parasitic nematodes (PPNs) are one of the most destructive pests in agriculture worldwide. PPNs with high virulence are widely spread in a broad range of commercially important crop families, such as Solanaceae, Fabaceae, Malvaceae, Amaranthaceae, and Poaceae. Furthermore, PPNs can survive in the soil for a long time before infesting again when suitable hosts emerge. Therefore, PPNs are difficult to control. As a result, they pose a large threat to the safety of global agricultural production [1]. As the most damaging nematodes in the family Heteroderidae, cyst nematodes cause huge annual yield losses globally. For instance, soybean cyst nematode (SCN, *Heterodera glycines*), a destructive pathogen in soybean (*Glycine max* (L.) Merr.) production worldwide, causes more than USD 1.5 billion of yield losses annually in the United States alone [2,3,4]. Currently, the most effective, economical, and environmentally friendly measure to control this pathogen is planting resistant soybean varieties. It is therefore important but challenging to map loci and clone the genes underlying SCN resistance for molecular breeding.

So far, the two major resistant genes in SCN-resistant quantitative trait loci (QTL), *rhg1* and *Rhg4*, in soybean have already been cloned and functionally identified. The resistant *rhg1* locus contains two types: Peking-type *rhg1-a* and PI 88788-type *rhg1-b* [5]. The *rhg1-b* carrying three resistant genes [*GmAAT*, *rhg1-b GmSNAP18* (an *α-SNAP*, *Glyma.18g022500*), and *GmWI12*] in a genomic segment of about 31 kb with multiple copies is solely required for SCN resistance of PI88788-type soybeans [6,7,8,9], while both *rhg1-a GmSNAP18* and *Rhg4* are needed for SCN resistance of Peking-type soybeans [10,11,12]. *GmSHMT08* (*Glyma.08g108900*), encoding a serine hydroxymethyltransferase, is the *Rhg4* gene on chromosome 08 [11]. In addition, *GmSNAP11* (*Glyma.11g234500*) on chromosome 11 has also been identified as a minor gene for SCN resistance of soybeans [13,14]. 

*GmSNAP18* on *rhg1* plays an important role in the cyst nematode resistance of soybeans. In resistant soybean varieties infected by SCN, GmSNAP18 would be abnormally accumulated in the feeding sites (syncytia), which showed cytotoxicity to cells, while the soybean NSF_Ran07_ could balance such cytotoxicity to maintain not only plant growth but also SCN resistance [15,16]. Recently, two syntaxins (Glyma.12g194800 and Glyma.16g154200) were reported to be able to target GmSNAP18 to mediate soybean SCN resistance [17]. A new Qa-SNARE protein, GmSYP31A, could interact with GmSNAP18 to regulate mitochondrial membrane signaling, thereby inducing cell death at SCN feeding sites and modulating resistance against SCN [18]. *GmSHMT08* impacted one-carbon folate metabolism by mediating soybean SCN resistance [11,19]. *Rhg4* also showed tandem repeats of a genomic segment of about 35.7 kb, which contains three genes: *Glyma.08g108800*, *GmSHMT08*, and *Glyma.08g109000* [20]. The pathogenesis-related protein GmPR08-Bet VI (Glyma.08g2320500) was involved in the resistance of soybean to SCN through interactions with both GmSNAP18 and GmSHMT08 [21]. However, the resistance mechanisms of *GmSNAP18* and *GmSHMT08* are still poorly known.

Butler et al. (2019) reported that overexpression of the *rhg1-b* carrying those three SCN-resistant genes in Arabidopsis and potato inhibited root and tuber growth, while enhancing resistance to beet cyst nematode (BCN, *Heterodera schachtii*) and potato cyst nematode (PCN, *Globodera rostochiensis*) [22]. However, neither *rhg1-a* GmSNAP18 nor *Rhg4* GmSHMT08 have been extended to other plant species for application in cyst nematode management. Our recent work studied whether *rhg1-a GmSNAP18* and *Rhg4 GmSHMT08*, in addition to their orthologs in Arabidopsis, *AtSNAP2* (an *α-SNAP*, *At3g56190*) and *AtSHMT4* (*At4g13930*), also conferred resistance to BCN using transgenic Arabidopsis. The obtained results revealed the opposite BCN-infection phenotypes of Arabidopsis between overexpressing *GmSNAP18* and *AtSNAP2*, and between overexpressing *GmSHMT08* and *AtSHMT4*: overexpression of either *GmSNAP18* or *GmSHMT08* enhanced BCN susceptibility of Arabidopsis, while overexpression of either *AtSNAP2* or *AtSHMT4* could suppress the susceptibility of Arabidopsis to BCN [23]. However, the resistance or susceptibility mechanisms of these *α-SNAPs* and *SHMTs* against BCN are unknown. In this study, we obtained the transgenic Arabidopsis simultaneously overexpressing *rhg1-a GmSNAP18* and *Rhg4 GmSHMT08*, evaluated their BCN-infection phenotypes, and analyzed their susceptibility mechanism against BCN together with the previously reported data.

## 2. Results

### 2.1. Simultaneous Overexpression of GmSNAP18 and GmSHMT08 Suppressed the Growth of Arabidopsis

**Figure 1:**
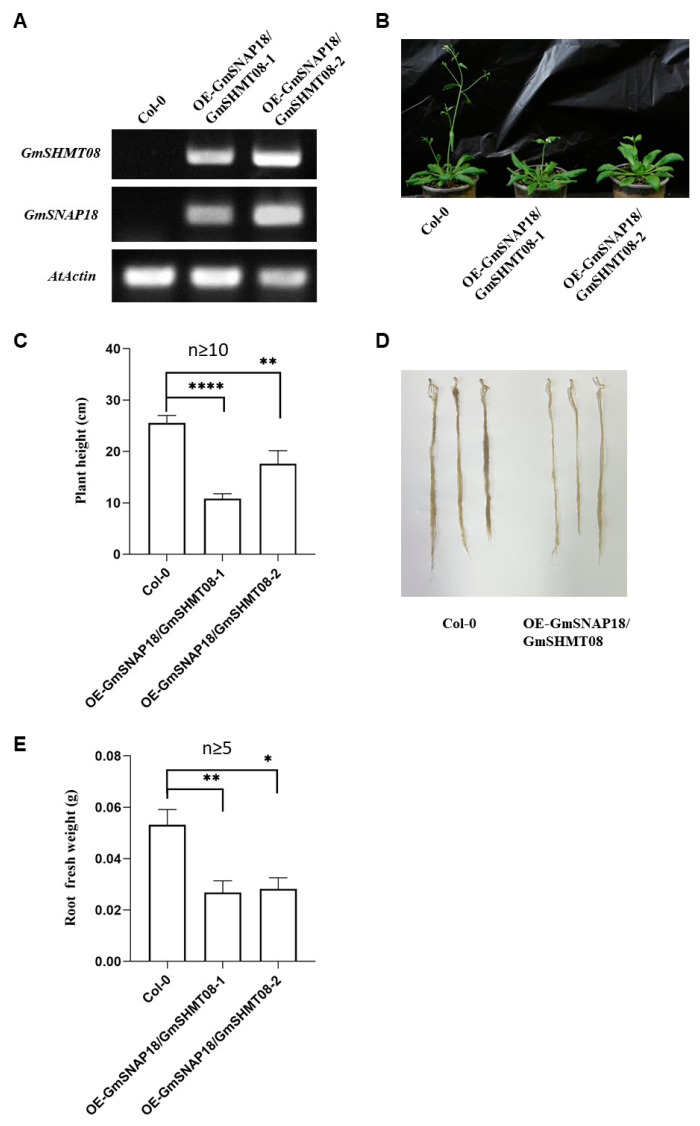
Effect of simultaneous overexpression of *GmSNAP18* and *GmSHMT08* on the growth of Arabidopsis. (**A**) Identification of the transgenic Arabidopsis lines (OE-GmSNAP18/GmSHMT08-1 and OE-GmSNAP18/GmSHMT08-2) by RT-PCR; Col-0: Wild-type Arabidopsis Col-0. (**B**) Pictures of the wild-type and transgenic plants 45 days after planting. (**C**) Statistics of plant height of the wild-type and transgenic Arabidopsis 45 days after planting (n ≥ 10). (**D**) Pictures of the roots of wild-type (Col-0) and transgenic plants 45 days after planting; (**E**): Effect on the fresh root weight of plants 45 days after planting (n ≥ 5). The significant difference was statistically analyzed by the one-way ANOVA method using the software Graphpad 8.0. *, *p* < 0.05; **, *p* < 0.01; ****, *p* < 0.0001.

Overexpression of *rhg1-a GmSNAP18* (hereafter used as *GmSNAP18*) impacted neither plant height nor root length, while overexpression of *GmSHMT08* stimulated plant height but did not affect root length in Arabidopsis [23]. In this study, we harvested seeds of two homologous T2 generation transgenic Arabidopsis lines simultaneously overexpressing *GmSNAP18* and *GmSHMT08*, OE-GmSNAP18/GmSHMT08-1 and OE-GmSNAP18/GmSHMT08-2 (Figure 1A), whose T3 generation plants were then used for the following measurements and analyses, including BCN-infection phenotyping. Concurrent overexpression of *GmSNAP18* and *GmSHMT08* substantially suppressed plant height when compared to wild-type Arabidopsis Col-0 (n ≥ 10) (Figure 1B,C). No significant difference in root length was shown between the transgenic Arabidopsis simultaneously overexpressing *GmSNAP18* and *GmSHMT08* and wild-type Arabidopsis Col-0 (Figure 1D). However, the fresh root weight of the transgenic Arabidopsis was remarkably decreased compared to wild-type Arabidopsis Col-0 (n ≥ 5) (Figure 1E). These results indicated that simultaneous overexpression of *GmSNAP18* and *GmSHMT08* suppressed the growth of both above-ground and under-ground parts of the transgenic Arabidopsis, different from individual overexpression of either *GmSNAP18* or *GmSHMT08* in Arabidopsis [23]. 

### 2.2. Simultaneous Overexpression of GmSNAP18 and GmSHMT08 Enhanced Susceptibility of Arabidopsis to BCN

**Figure 2:**
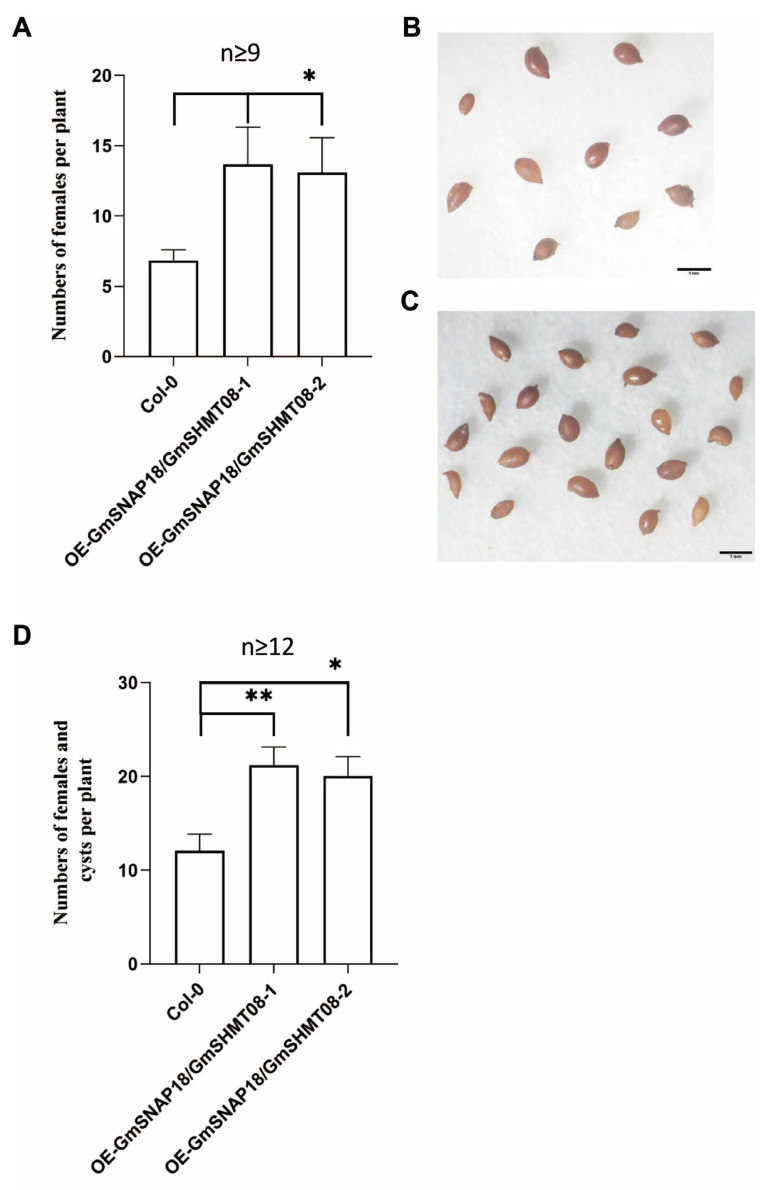
Phenotyping of *GmSNAP18* and *GmSHMT08*-simultaneously overexpressed Arabidopsis infected by BCN. (**A**) Statistics of *GmSNAP18* and *GmSHMT08*-simultaneously overexpressed Arabidopsis at 20 days post-inoculation (dpi) of BCN (n ≥ 9). (**B**) Cysts in a wild-type Arabidopsis Col-0 plant at 35 dpi on average. (**C**) Cysts in a *GmSNAP18* and *GmSHMT08*-simultaneously overexpressed Arabidopsis plant at 35 dpi on average. (**D**) Statistics of *GmSNAP18* and *GmSHMT08*-simultaneously overexpressed Arabidopsis at 35 dpi of BCN (n ≥ 12). The significant difference was statistically analyzed by the one-way ANOVA method using the software Graphpad 8.0. *, *p* < 0.05; **, *p* < 0.01.

Subsequently, the BCN-infection phenotypes of transgenic Arabidopsis simultaneously overexpressing *GmSNAP18* and *GmSHMT08* were evaluated. Clearly, at 20 days post-inoculation (dpi) of BCN, the numbers of females per plant simultaneously overexpressing *GmSNAP18* and *GmSHMT08* were substantially increased when compared to wild-type Arabidopsis Col-0 (n ≥ 9) (Figure 2A). At 35 dpi, compared to wild-type Arabidopsis Col-0, the total numbers of both females and cysts per plant simultaneously overexpressing *GmSNAP18* and *GmSHMT08* were also significantly elevated (n ≥ 12) (Figure 2B–D). As stated above, simultaneous overexpression of *GmSNAP18* and *GmSHMT08* inhibited the root growth of Arabidopsis (Figure 1D,E), so the BCN-infection phenotype of the transgenic Arabidopsis is unrelated to root growth status. It could therefore be concluded from these obtained results that simultaneous overexpression of *GmSNAP18* and *GmSHMT08* boosted the susceptibility of Arabidopsis to BCN.

### 2.3. Simultaneous Overexpression of GmSNAP18 and GmSHMT08 Suppressed the Expression Patterns of AtPR1 on the Salicylic Acid Signaling Pathway in Arabidopsis

**Figure 3:**
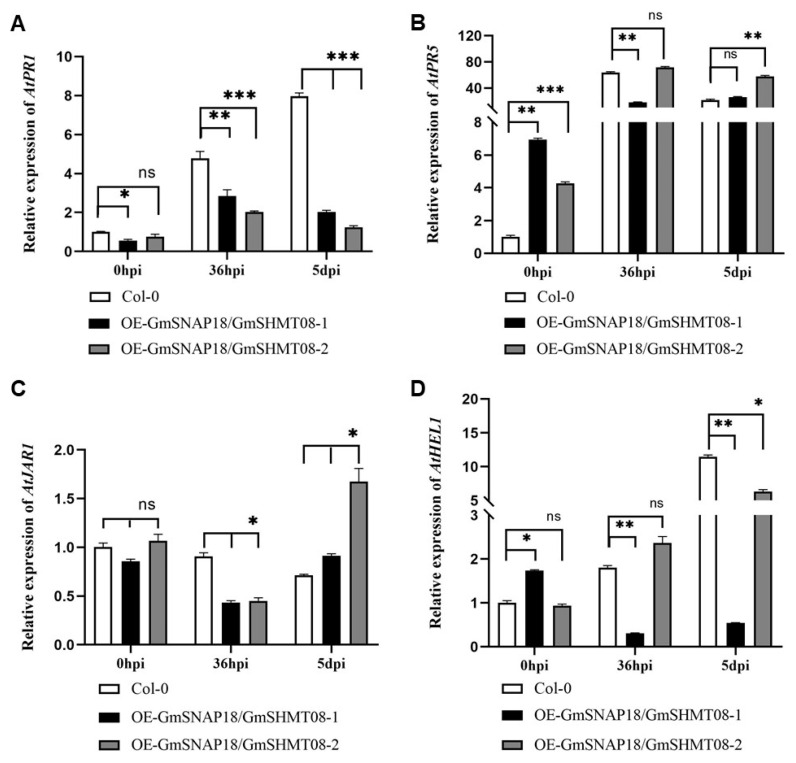
Expression patterns of *AtPR1*, *AtPR5*, *AtJAR1*, and *AtHEL1* in *GmSNAP18* and *GmSHMT08*-simultaneously overexpressed Arabidopsis infected by BCN. (**A**) Expression patterns of *AtPR1*. (**B**) Expression patterns of *AtPR5*. (**C**) Expression patterns of *AtJAR1*. (**D**) Expression patterns of *AtHEL1*. The relative expression levels were obtained after comparing them to those in the wild-type plants at 0 hpi, which was set as ‘1′. The experiments were repeated three times, with a similar trend. The significant difference was statistically analyzed by the one-way ANOVA method using the software Graphpad 8.0. ns: No significance; *, *p* < 0.05; **, *p* < 0.01; ***, *p* < 0.001.

Arabidopsis AtPR1 (At2g14610) rather than AtPR5 (At1g75040) interacted with AtSNAP2, AtSHMT4, and the BCN effector HsSNARE1, which was involved in mediating BCN susceptibility [24]. In this work, the expression patterns of both *AtPR1* and *AtPR5* on the salicylic acid (SA) signaling pathway in the transgenic Arabidopsis simultaneously overexpressing *GmSNAP18* and *GmSHMT08* were analyzed. The results clearly indicated that overexpression of both *GmSNAP18* and *GmSHMT08* substantially suppressed expression of *AtPR1*; in contrast, overexpression of both *GmSNAP18* and *GmSHMT08* did not remarkably impact expression of *AtPR5*, in the transgenic Arabidopsis when compared to wild-type Arabidopsis Col-0, after infected by BCN, no matter at 36 h post-inoculation (hpi) or 5 dpi (Figure 3A,B). 

### 2.4. Simultaneous Overexpression of GmSNAP18 and GmSHMT08 Did Not Impact Expression Patterns of AtJAR1 and AtHEL1 on the Jasmonic Acid and Ethylene Signaling Pathways in Arabidopsis

Subsequently, we studied whether simultaneous overexpression of *GmSNAP18* and *GmSHMT08* impacted the expression patterns of *AtJAR1* (*At2g46370*) and *AtHEL1* (*At3g04720*) on the jasmonic acid (JA) and ethylene (ET) signaling pathways in Arabidopsis. The results showed that, compared to wild-type Arabidopsis Col-0, expression patterns of neither *AtJAR1* nor *AtHEL1* showed similar trends in both transgenic lines at 5 dpi (Figure 3C,D). Thus, expression patterns of *AtJAR1* and *AtHEL1* were not associated with *GmSNAP18* and *GmSHMT08* expression in Arabidopsis, meaning simultaneous overexpression of *GmSNAP18* and *GmSHMT08* might not impact the JA and ET signaling pathways.

### 2.5. Subcellular and Interaction Localizations of AtSNAP2, AtSHMT4, and AtPR1

**Figure 4:**
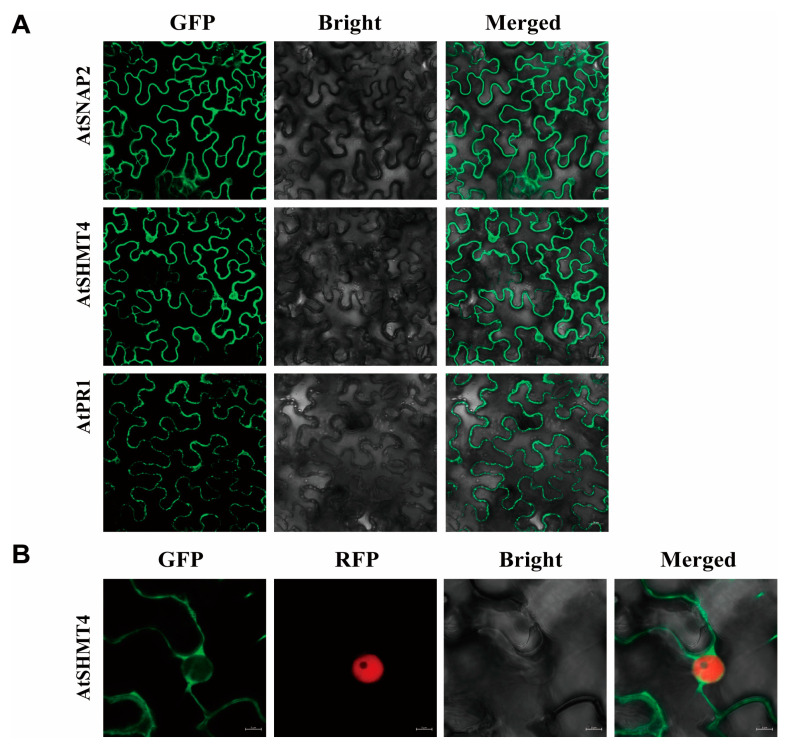
Subcellular localizations of AtSNAP2, AtSHMT4, and AtPR1 in *Nicotiana benthamiana* cells. RFP: H2B-RFP nucleus signal marker. (**A**) Subcellular localizations of AtSNAP2, AtSHMT4, and AtPR1. (**B**) Subcellular localization of AtSHMT4 with nucleus signal marker.

**Figure 5:**
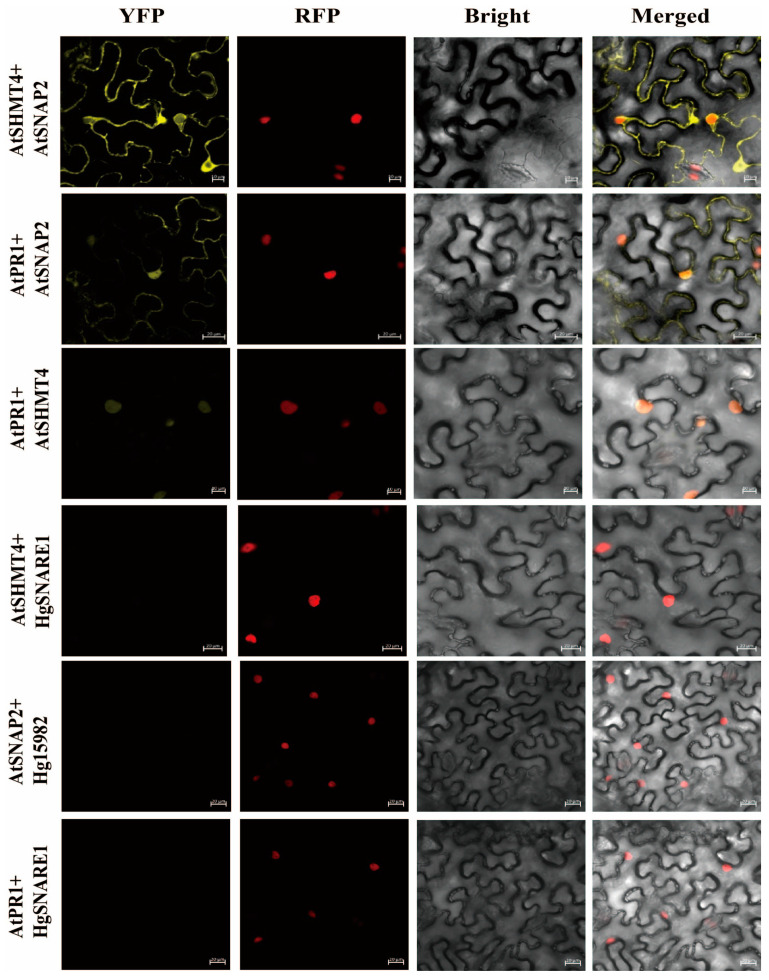
Interaction localizations of AtSNAP2, AtSHMT4, and AtPR1 in *Nicotiana benthamiana* cells by BiFC assay. RFP: H2B-RFP nucleus signal marker. AtSHMT4 + HgSNARE1 (Hetgly.T0000011771.1), AtSNAP2 + Hg15982 (Hetgly.T0000015982.1), and AtPR1 + HgSNARE1 were used as the negative controls.

The subcellular localization analyses showed that both AtSNAP2 and AtPR1 were localized on the plasma membrane of cells, while AtSHMT4 was localized in the cell nucleus besides on the plasma membrane of cells of *Nicotiana benthamiana* (Figure 4A,B). AtSNAP2, AtSHMT4, and AtPR1 could interact pair-wisely [24]. We further analyzed the localization of their interactions. The BiFC assays indicated that interactions between AtSNAP2 and AtSHMT4, and between AtPR1 and AtSNAP2 could occur both on the plasma membrane and in the nucleus, while AtPR1 and AtSHMT4 could interact only in the nucleus of *Nicotiana benthamiana* cells (Figure 5), suggesting the translocation of AtSNAP2 and AtPR1 into the nucleus of cells after interactions due to AtSHMT4. 

## 3. Discussion

*Rhg4* and *rhg1* (*rhg1-a* and *rhg1-b*) are two major QTL underlying SCN resistance in soybean [10,25]. Both *rhg1-a* and *Rhg4* are required for the SCN resistance of Peking-type soybeans, while *rhg1-b* is solely needed for the SCN resistance of PI 88788-type soybeans [5,10,25]. *GmSNAP18* and *GmSHMT08* are the resistant genes on *rhg1-a* and *Rhg4*, respectively [11,12]. In conjunction with our previous study [23], this present work studied the possibility of extension application of SCN-resistant *rhg1-a GmSNAP18* and *Rhg4 GmSHMT08* for management of cyst nematodes by simultaneously expressing them into Arabidopsis infected by BCN. However, overexpression of both *rhg1-a GmSNAP18* and *Rhg4 GmSHMT08* (Figure 2) or either of them [23] enhanced BCN susceptibility of Arabidopsis. These indicate different mechanisms of resistance and susceptibility of *rhg1-a GmSNAP18* and *Rhg4 GmSHMT08* to SCN and BCN in soybean and Arabidopsis, respectively. In contrast, overexpression of either *AtSNAP2* or *AtSHMT4*, which are the orthologs of *rhg1-a GmSNAP18* and *Rhg4 GmSHMT4* in Arabidopsis, respectively, suppressed BCN susceptibility [23]. 

**Figure 6:**
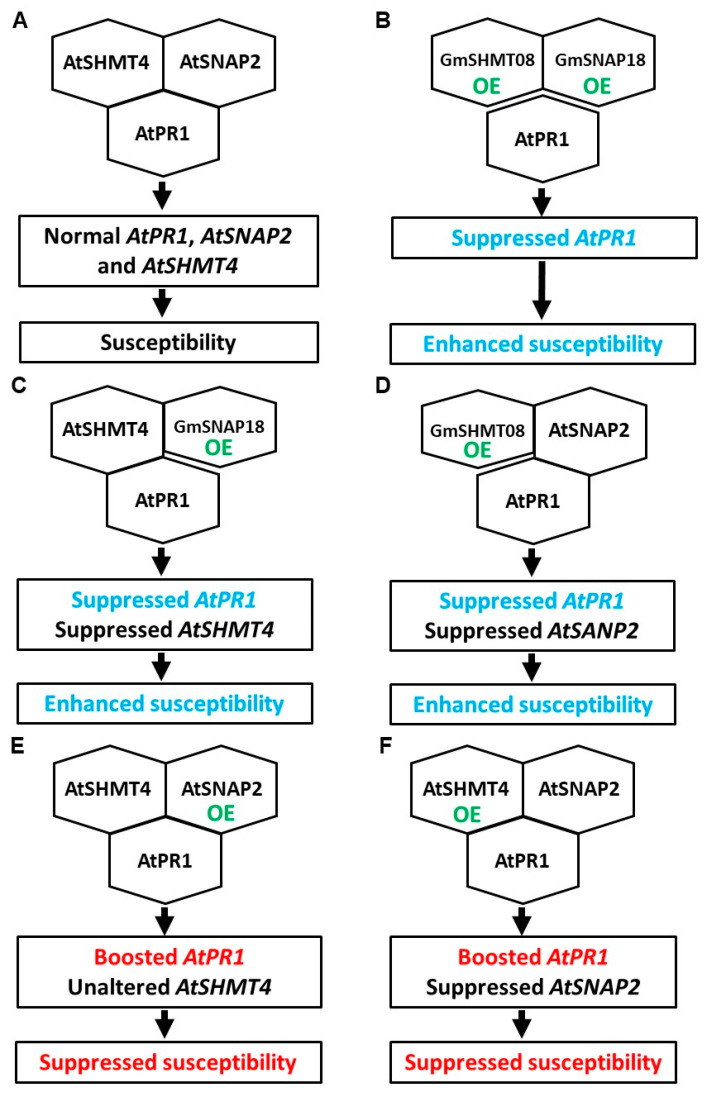
Hypothesized molecular models of action for mediation of BCN susceptibility of Arabidopsis. (**A**–**F**) Hypothesized molecular models of action for mediation of BCN susceptibility of wild-type Arabidopsis Col-0 and transgenic Arabidopsis overexpressing both *GmSNAP18* and *GmSHMT08* or either of them, or either of *AtSNAP2* or *AtSHMT4*. The models denote that AtSNAP2, AtSHMT4, and AtPR1 interacted pair-wisely in (**A**,**E**,**F**), but GmSNAP18/AtSNAP2, GmSHMT08/AtSHMT4, and AtPR1 did not interact pair-wisely in (**B**–**D**). OE: Overexpression.

GmSNAP18, GmSHMT08, and GmPR08-Bet VI in soybean, and AtSNAP2, AtSHMT4, and AtPR1 in Arabidopsis could interact pair-wisely [21,24]. A simple hypothesized molecular model of action for wild-type Arabidopsis Col-0 is shown in Figure 6A. GmSNAP18 interacted with AtSHMT4, and GmSHMT4 interacted with AtSNAP2; however, neither GmSNAP18 nor GmSHMT08 interacted with AtPR1 [23]. Thus, no pairwise interactions among GmSNAP18, GmSHMT08, and AtPR1 occurred in Arabidopsis overexpressing either *GmSNAP18* or *GmSHMT08*, or both of them. When compared to wild-type Arabidopsis Col-0, the transgenic Arabidopsis overexpressing both *GmSNAP18* and *GmSHMT08* substantially suppressed *AtPR1* expression (Figure 3A), similar to the transgenic Arabidopsis overexpressing either *GmSNAP18* or *GmSHMT08* [23]. Additionally, overexpression of either *AtSNAP2* or *AtSHMT4* substantially suppressed BCN susceptibility and remarkably enhanced *AtPR1* expression in the transgenic Arabidopsis compared to wild-type Arabidopsis Col-0 [23]. We thus hypothesized the simple models of action for different types of α-SNAPs, SHMTs, and AtPR1 in the mediation of BCN susceptibility in Arabidopsis (Figure 6B–F). Taken together, no pairwise interactions of GmSNAP18, GmSHMT08, and AtPR1 with suppressed *AtPR1* expression enhanced BCN susceptibility in Arabidopsis.

Expression of *AtPR1* on the SA signaling pathway would be suppressed in transgenic Arabidopsis overexpressing both *rhg1-a GmSNAP18* and *Rhg4 GmSHMT08* or either of them; in contrast, *AtPR1* expression would be stimulated in transgenic Arabidopsis overexpressing either *AtSNAP2* or *AtSHMT4* (Figure 4; ref. [23]). However, the expression pattern of *AtPR5* was not impacted by the simultaneous overexpression of *GmSNAP18* and *GmSHMT08* in Arabidopsis after infection with BCN (Figure 3B). Furthermore, the expression pattern of neither *AtJAR1* nor *AtHEL1* on the JA and ET signaling pathways was influenced by the simultaneous overexpression of *GmSNAP18* and *GmSHMT08* in Arabidopsis after infection with BCN (Figure 3C,D). In addition, cytokinins were reported to be involved in plant-pathogen interactions [26], but to the best of our knowledge, there are rarely reports about cytokinins in the mediation of soybean cyst nematode resistance. So, in this study, we did not measure the expression patterns of cytokinins in the transgenic Arabidopsis simultaneously overexpressing *GmSNAP18* and *GmSHMT08*. These suggest BCN susceptibility of Arabidopsis may be mainly associated with the SA signaling pathway. Translocations of AtSNAP2 and AtPR1, both of which were localized on the plasma membrane, into the nucleus occurred in *Nicotiana benthamiana* cells after interactions due to AtSHMT4, which was localized both on the plasma membrane and in the nucleus of cells (Figure 4 and Figure 5). Therefore, *AtPR1* expression is mediated by the interactions of AtPR1 with AtSNAP2 and AtSHMT4 and the interaction between AtSNAP2 and AtSHMT4; while such pair-wise interactions are broken down, *AtPR1* expression will be suppressed, as shown in the case of simultaneous overexpression of *GmSNAP18* and *GmSHMT08* in Arabidopsis (Figure 3A). Pathogenesis-related (*PR*) genes are one key component in the SA signaling pathway, which play an important role in plant-pathogen interactions and are particularly essential for regulating the resistance of plants to pathogens, including nematodes. Tomato PR-1 was a hallmark of the cultivar resistance against PCN conferred by the resistant gene *Hero A* [27]. Tomato pathogenesis-related genes, particularly *PR-1*, were markedly involved in *Mi-1*-mediated and SA-induced resistance to root-knot nematodes (*Meloidogyne incognita*) [28]. The resistance to SCN in soybean would be enhanced by overexpressing *AtPR5* in susceptible soybean Williams 82 [29]. In addition, GmPR08-Bet VI could interact with both *Rhg4* GmSHMT08 and *rhg1-a* GmSNAP18 to be involved in mediating SCN resistance in Peking-type soybeans [21,30]. Recently, our study revealed a novel mechanism for mediating BCN resistance of Arabidopsis via AtPR1: a BCN effector HsSNARE1 could interact with AtPR1 and AtSNAP2 via its N-terminal and t-SNARE domain, respectively, to form a super-complex composed of HsSNARE1, AtPR1, AtSNAP2, and AtSHMT4, which suppressed the expression of *AtPR1* and ultimately promoted nematode parasitism [24]. These combined comparisons further support that no pairwise interactions of GmSNAP18, GmSHMT08, and AtPR1 with suppressed expression of *AtPR1* enhanced the susceptibility of Arabidopsis to BCN. Nuaima et al. (2023) studied six *Heterodera schachtii* populations that coincided with differences in invasion and propagation in plant roots, which show that the plant–nematode interaction between cruciferous plants and *H. schachtii* occurred in a host- and population-specific manner [31]. The specialized interaction with each plant variety may explain why GmSNAP18 and GmSHMT08 show different interactions in soybean and Arabidopsis with SCN and BCN.

However, overexpression of soybean *rhg1-b* carrying 3 resistant genes (*rhg1-b GmSNAP18*, *GmAAT*, and *GmWI12*; ref. [6]) suppressed BCN susceptibility in Arabidopsis [22], in contrast to simultaneous overexpression of *rhg1-a GmSNAP18* and *Rhg4 GmSHMT08* in Arabidopsis (Figure 2). Cyst nematode resistance of *rhg1-b* in both soybean and Arabidopsis may not require involvement of the PRs on the SA signaling pathway; in contrast, cyst nematode resistance and susceptibility of *rhg1-a GmSNAP18* and *Rhg4 GmSHMT08* in soybean and Arabidopsis, respectively, are essentially associated with the PRs-related SA signaling pathway, which is worthy of further study. As we know, rational control of the nematodes is critical to helping improve crop yields globally. As a result, exploiting plant resistance and the molecular mechanisms underlying plant–nematode interactions is key when materializing the impacts on a case-by-case basis [32]. This will help us optimize PPN control by combining them with other tactics in integrated management.

## 4. Materials and Methods

### 4.1. Plant Materials and Nematodes

Arabidopsis Col-0 was used as the wild-type. Arabidopsis plants were grown under long-day conditions (16 h light/8 h dark cycles) at 24 °C. *Nicotiana benthamiana* was planted in soil and grew under a 16 h light/8 h dark photoperiod at 24–25 °C. BCN was used as the nematode and propagated on beets (*Beta vulgaris* L.) [23]. 

### 4.2. Gene Cloning and Plasmid Construction

For the construction of transgenic Arabidopsis, cDNAs of *rhg1-a GmSNAP18* and *GmSHMT08* were, respectively, cloned into pH7WG2D and pDT7 with a CaMV35S promoter (pCaMV35S) to generate pH7WG2D: *rhg1-a GmSNAP18* and pDT7:*GmSHMT08* using a ClonExpress II One Step Cloning kit (Vazyme, Nanjing, China). cDNAs of *rhg1-a GmSNAP18* and *GmSHMT08* were cloned from the soybean cultivar (*cv.*) Forrest, which shows Peking-type SCN resistance, using PrimeSTAR^®^ Max DNA Polymerase (Takara, Kusatsu, Japan). Total RNA was extracted with a TRIzol^TM^ Reagent (Invitrogen, Vilnius, Lithuania), and the cDNA was synthesized using a HiScript III 1st Strand cDNA Synthesis Kit (+gDNA wiper) (Vazyme, Nanjing, China).

For the subcellular localization, *AtSNAP2*, *AtSHMT4,* and *AtPR1* were amplified using the corresponding primers listed in Table 1 and cloned into pYBA1132 fused with a GFP at the C-terminus to generate pYBA1132:AtSNAP2, pYBA1132:AtSHMT4, and pYBA1132:AtPR1.

For the BiFC assay, *AtSNAP2*, *AtSHMT4,* and *AtPR1* were cloned into pSPYNE(R)173 to generate pSPYNE(R)173:AtSNAP2, pSPYNE(R)173:AtSHMT4, and pSPYNE(R)173:AtPR1. AtSHMT4 and AtPR1 were cloned into pSPYCE(M) to generate pSPYCE(M):AtSHMT4 and pSPYCE(M):AtPR1, respectively. The primers are listed in Table 1. 

### 4.3. Arabidopsis Transformation and Molecular Identification

The two constructs pH7WG2D:*rhg1-a* GmSNAP18 and pDT7:GmSHMT08 were first, respectively, transformed into *Agrobacterium tumefaciens* GV3101 using the freeze–thaw method. Subsequently, Arabidopsis transformation was conducted by the flower bud soaking method [33]. The transformed seedlings were drained a little and grew for 24 h in the dark and then under the normal growth conditions of long-day conditions (16 h light/8 h dark cycles) at 24 ℃. The harvested T1 seeds were screened on the 1/2 MS medium with BASTA and hygromycin to obtain positive seedlings, which were further identified by RT-PCR using the corresponding primers listed in Table 1, generating a *rhg1-a GmSNAP18* fragment of 119 bp, and a *GmSHMT08* fragment of 252 bp. *AtActin* (At5g09810) was used as the reference gene. The positive seedlings were transferred into soils to grow and harvest T2 seeds for each plant as the transgene lines (OE-GmSNAP18/GmSHMT08). The homologous T3 generation plants were used for analyses including growth status, BCN-infection phenotyping, gene expression patterns.

### 4.4. Growth Parameter Measurement of Transgenic Arabidopsis

At least 20 seedlings each transgenic Arabidopsis line (OE-GmSNAP18/GmSHMT08-1 and OE-GmSNAP18/GmSHMT08-2) were planted in the soil for measurement of growth parameters, including plant height, root length, and fresh root weight. The plants grown in the soil were measured about 45 days after planted. The experiments were repeated three batches with a similar experimental trend each batch. The significant difference was statistically analyzed by one-way ANOVA method using the software Graphpad 8.0.

### 4.5. Phenotyping of Arabidopsis Infected with BCN

Two identified homozygous transgenic Arabidopsis lines, OE-GmSNAP18/GmSHMT08-1 and OE-GmSNAP18/GmSHMT08-2, with infection of BCN were phenotyped using the method of Zhao and Liu (2023) [24] with minor modifications. Briefly, the wild-type and transgenic Arabidopsis seeds were planted in the soil about 10 days after germination by spraying BASTA. The obtained positive seedlings were transplanted into the plastic cups filled with sand and soil (7:3, *w*/*w*) and grew for 3–4 weeks at 24 °C under 16 h/8 h light/dark conditions. Then, each seedling was inoculated with 400 hatched BCN J2s. The samples, including the seedlings and soils, were collected at 20 and 35 dpi to observe and count BCN females and cysts under an Olympus SE61 stereomicroscope (Olympus, Tokyo, Japan). The experiments were conducted independently for three batches with at least 9 replicates each line each batch. The significant difference was statistically analyzed by one-way ANOVA method using the software Graphpad 8.0.

### 4.6. Quantitative Real-Time PCR

The expression patterns of *AtPR1*, *AtPR5*, *AtJAR1* and *AtHEL1* in Arabidopsis were analyzed using quantitative real-time PCR (qRT-PCR). Each Arabidopsis seedling was inoculated with 400 BCN J2s. Roots were collected at 0 h, 36 h, and 5 days post-inoculation (hpi/dpi), respectively. The mRNA was extracted from the collected Arabidopsis roots at different time-frame points employing a Dynabeads mRNA DIRECT kit (Invitrogen, Vilnius, Lithuania), and the cDNA was synthesized using a PrimeScript™ RT reagent kit with gDNA Eraser kit (Takara, Kusatsu, Japan). The qRT-PCR reaction solutions were prepared using a TB Green^TM^ Premix Ex Taq^TM^ (Tli RNaseH Plus) kit (Takara, Kusatsu, Japan), and the qRT-PCR was conducted on a 7500 Fast Real-Time PCR system (Applied Biosystems, Waltham, MA, USA). *AtActin* was used as the reference gene. The corresponding primers are listed in Table 1. The relative expression was calculated relative to the expression level of wild-type Col-0 before inoculation (0 hpi), which was set as ‘1′, by the 2^−ΔΔCt^ method [34]. Three replicates were set each time for these experiments, and the experiments were replicated three times. The significant difference in gene expression in the transgenic Arabidopsis relative to the wild-type Col-0 at the same time-frame point was statistically analyzed by one-way ANOVA method using the software Graphpad 8.0. 

### 4.7. Subcellular Localization and BiFC Assay

Subcellular localization and BiFC assay were performed as previously described [35,36]. The plasmids including pYBA1132:AtSNAP2, pYBA1132:AtSHMT4 and pYBA1132:AtPR1 for the subcellular localization analysis and pSPYNE(R)173:AtSNAP2, pSPYNE(R)173:AtSHMT4, pSPYNE(R)173:AtPR1, pSPYCE(M):AtPR1 and pSPYCE(M):AtSHMT4 for BiFC assay were transformed into *Agrobacterium tumefaciens* strain EHA105 competent cells to prepare Agrobacterium suspensions, which were resuspended in infiltration buffer [10 mmol L^−1^ MgCl_2_ and 10 mmol L^−1^ MES (pH 5.6) and 200 μmol L^−1^ acetosyringone]. The suspensions were infiltrated into *Nicotiana benthamiana* leaves using a 1 mL syringe after 3 h incubation at room temperature. The 4–5 leaf stage wild-type *Nicotiana benthamiana* plants were used for Agrobacterium-mediated transient expression. At 36–48 hpi, the fluorescence was observed under a Zeiss LSM 980 laser confocal microscope (Carl Zeiss LSM T-PMT, Oberkochen, Germany). GFP was excited at 488 nm and captured at 510–550 nm; YFP was excited at 514 nm and captured at 565–585 nm; RFP was excited at 543 nm and captured at 590–630 nm. The collected images were processed using ZEN 2 (Carl Zeiss Microscope GmbH2011).

## Figures and Tables

**Table 1:** List of the primers used in this study.

Name	Primer Sequence (5’-3’)
**Primers for cloning of *rhg1-a GmSNAP18* and *GmSHMT08***	
*rhg1-a* GmSNAP18 (Glyma.18g022500)	F: ATGGCCGATCAGTTATCGAAGGG
	R: TCAAGTAATAACCTCATACTCCTCA
GmSHMT08 (Glyma.08g108900)	F: ATGGATCCAGTAAGCGTGTGG
	R: CTAATCCTTGTACTTCATTTCAG
**Primers for plasmid construction**	
*rhg1-a* GmSNAP18	F: TGTGACCTCGAGACTAGTATGGCCGATCAGTTATCGAAGGG
	R: CCGTCGCACCATACTAGTAGTAATAACCTCATACTCCTCAAG
GmSHMT08	F: TGTGACCTCGAGACTAGTATGGATCCAGTAAGCGTGTGG
	R: CCGTCGCACCATACTAGTATCCTTGTACTTCATTTCAG
**Primers for identification of transgenic Arabidopsis**	
*rhg1-a* GmSNAP18	F: CAAGCTCGCCAAATCATGGG
	R: AGCAATGTGCAGCATCGACA
GmSHMT08	F: ATGGATCCAGTAAGCGTGTGGGGTA
	R: TGAGCGGCAGAGGTTTTCG
AtActin (At5g09810)	F: GCATGAAGATCAAGGTGGTTGCAC
	R: ATGGACCTGACTCATCGTACTCACT
**Primers for gene expression analyses**	
AtPR1 (At2g14610)	F: ACGGGGAAAACTTAGCCTGG
	R: TTGGCACATCCGAGTCTCAC
AtPR5 (At1g75040)	F: AGGCTGCAACTTTGACGC
	R: AGAAATCTTTGCCGCCATC
AtHEL1 (At3g04720)	F: GATAAGCCGTACGCATGGC
	R: TCACCCTTAAACACTTGCCG
AtJAR1 (At2g46370)	F: GCTACATTTGCTGTGATTCCG
	R: GGTATCGATACAACCCTGCG
AtActin	F: GCATGAAGATCAAGGTGGTTGCAC
	R: ATGGACCTGACTCATCGTACTCACT
**Primers for bimolecular fluorescence complementation (BiFC)**	
pSPYNE(R)173-AtSNAP2	F: AGGCCTACTAGTGGATCCATGGGGGATCATCTGGTGAG
	R: TTCGAGCTCCTACCCGGGTCATGTAAGGTCATCCTCCTCTAG
pSPYNE(R)173-AtPR1	F: AGGCCTACTAGTGGATCCATGAATTTTACTGGCTATTC
	R: TTCGAGCTCCTACCCGGGTTAGTATGGCTTCTCGTTCACA
pSPYCE(M)-AtPR1	F: ACTAGTGGATCCATCGATATGAATTTTACTGGCTATTC
	R: GTATGGGTACATCCCGGGGTATGGCTTCTCGTTCACA
pSPYNE(R)173-AtSHMT4	F: AGGCCTACTAGTGGATCCATGGAACCAGTCTCTTCATG
	R: TTCGAGCTCCTACCCGGGCTAATCCTTGTACTTCATCTC
pSPYCE(M)-AtSHMT4	F: ACTAGTGGATCCATCGATATGGAACCAGTCTCTTCATG
	R: GTATGGGTACATCCCGGGATCCTTGTACTTCATCTC
**Primers for subcellular localization**	
pYBA1132-AtPR1	F: TCTAGAACTAGTGGATCCATGAATTTTACTGGCTATTC
	R: GAGGTCGACGGTATCGATGTATGGCTTCTCGTTCACA
pYBA1132-AtSNAP2	F: TCTAGAACTAGTGGATCCATGGGGGATCATCTGGTGAG
	R: GAGGTCGACGGTATCGATTGTAAGGTCATCCTCCTCTAG
pYBA1132-AtSHMT4	F: TCTAGAACTAGTGGATCCATGGAACCAGTCTCTTCATG
	R: GAGGTCGACGGTATCGATATCCTTGTACTTCATCTC

## Data Availability

Data are contained within the article.
